# Effects of different preservation schemes on isolated rat artery

**DOI:** 10.1111/jcmm.17822

**Published:** 2023-06-25

**Authors:** Zhang‐Yong Ren, Qiao Wu, Bing Pan, Jia‐Zong Liu, Qiang He, Ren Lang, Shao‐Cheng Lyu

**Affiliations:** ^1^ Department of Hepatobiliary Surgery, Beijing Chao‐Yang Hospital Capital Medical University Beijing China; ^2^ Beijing Organ Transplant Center Beijing China

**Keywords:** cold storage, frozen storage, rat artery, safety, vascular preservation

## Abstract

Allogeneic blood vessels are regarded as one of the best natural substitutes for diseased blood vessels due to their good vascular compliance and histocompatibility. Since the supply and demand of allograft blood vessels do not always match in time and space, a good preservation scheme for isolated blood vessels is essential. The abdominal aortas of 110 male Sprague–Dawley (SD) rats were randomly divided into three groups, including cold storage group (4°C) (CSG), frozen storage group (FSG) and ambient storage group (25 ± 2°C) (ASG). Seven time points of preservation for 1, 3, 5, 7, 14, 30 and 90 days were set for detection. The changes in vascular physiological function were evaluated by MTT test and vasoconstriction ability detection, and the changes in vascular wall structure were evaluated by the tension tolerance test and pathological staining. The vascular function of CSG was better than FSG within first the 7 days, but the result was opposite since the 14th day. The vascular wall structure, collagen and elastic fibres of vessels, in CSG, showed oedema within 30 days, and continuous disintegration and rupture at 90 days. The vessel wall structure of FSG remained intact within 90 days. The tensile strength of the vessels in CSG was better than that in FSG within 5 days, and there was no statistical difference between the two groups between the 7th and 30th day, and then, the FSG was higher than CSG on the 90th day. Both cold storage and frozen storage could be applied as safe and effective preservation schemes for isolated rat artery within first 30 days. Cold storage is recommended when the storage time is <14 days, and then, frozen storage is better.

## 1 INTRODUCTION

With the continuous development of microsurgery and surgical technique, the technique of vascular reconstruction is gaining more and more popularity in clinical practice.^1^, ^2^, ^3^, ^4^ Currently, autologous vessels, artificial vessels and allogeneic vessels are the most commonly used vascular grafts for vascular reconstruction. However, the application of autologous vessels is limited due to its insufficient source, extended surgical trauma and application restrictions from diameter, length and the vascular condition of patients.^5^ Although artificial vessels have the advantages of adequate sources and variable diameter and length based on clinical needs, its poor biocapacity, high thrombosis rate and inability to replace small vessels are still puzzling medical researchers.^6^ As early as 1979, Starzl, et al.^7^ suggested preserving the allogeneic vessels from organ donors under cold storage condition in case of unexpected vascular problems during abdominal surgeries. With the development of organ donation, the sources of allogeneic vessels have been increasingly abundant. Considering its advantages in adequate sources, matched vascular diameter, well biocapacity, and high short‐term and long‐term patency rate, the application of allogeneic vessels in vascular reconstruction is promising.^8^, ^9^

However, fresh allogeneic vessels are not always available in clinical practice, urging the necessity of adopting proper preservation schemes to store these vessels for clinical needs effectively. An ideal vascular condition and proper procedure for vascular preservation might influence surgical safety and patients' prognosis directly.^10^, ^11^, ^12^ Cold storage at 4°C and frozen storage at −196°C are the most used schemes for vascular preservation in current clinical practice.^13^ Although it is generally believed that frozen storage at −196°C can more persistently and efficiently preserve the activity of cells and tissues, it requires cumbersome cooling and rewarming procedures, as well as expensive equipment. More researchers gradually begin to explore the applicational potential and advantages of cold storage in the short‐term preservation of isolated blood vessels.

In this study, the structure, cell activity, vascular motor function and anti‐tension of isolated abdominal aorta of SD rats using different preservation schemes were elevated and compared. The objective of the present study is to provide a reference for the selection of preservation schemes of isolated blood vessels in the clinic.

## 2 MATERIALS AND METHODS

### 2.1 Experimental material

#### 2.1.1 Experimental animals

A total of 110 Specific Pathogen Free male SD rats aged 6–8 weeks and weighing about 250 g were selected. All animals were purchased from Beijing Weitong Lihua Animal Experimental and housed in an animal room kept at a temperature of 22 ± 3°C with 55% ± 20% humidity, 6–20 air changes/h and a 12‐h light/dark cycle at the Medical Research Center of Beijing Chao‐Yang Hospital (Beijing, China). The animals were cared for and euthanized according to the principles outlined in the Guidelines for the Care and Use of Laboratory Animals by the Animal Experiment and Laboratory Animal Welfare Committee of Capital Medical University (No: AEEI‐2021‐147).

#### 2.1.2 Experimental instruments

Experimental instrument: Multi Myograph System‐610 M tension measuring instrument (Danish Myo Technology A hand S company), vascular tension computer software (LabChart Pro v7.3.8, AD Inscrurenl company), multi‐function enzyme label instrument (Varioskan Flash, Thermo Scientific company) and Cell Freezing Container (Corning).

#### 2.1.3 Experimental reagents

Phenylephrine (Shanghai Hefeng Pharmaceutical), Thiazolyl tetrazolium bromide (MTT, Shanghai Aladdin Biochemical Technology Co., Ltd.), Dimethyl Sulfoxide (DMSO, Sigma, Appendix S1), Medium 199 (Sigma), Penicillin–streptomycin (Semel Fisher Technology Co., Ltd.) and HEPES buffer (Gibco).

### 2.2 Experimental methods

#### 2.2.1 Blood vessel acquisition

Sprague–Dawley rats were anaesthetised by intraperitoneal injection of pentobarbital sodium (50 mg/kg), and the skin of abdomen was disinfected with 1% povidone‐iodine solution and 75% ethanol. The abdominal aorta with about 2 cm was obtained through a longitudinal incision and was rinsed with normal saline (NS) to eliminate the remaining blood stain. After a thorough observation under the microscope to confirm no residual blood cells or debris, the perivascular fat and small branches were removed under an anatomical microscope. The isolated abdominal aortas were stored according to the protocol of different groups.

#### 2.2.2 Experimental group

All aortas were randomly divided into three groups: the CSG, the FSG and the ASG. Seven time points of storage for 1, 3, 5, 7, 14, 30 and 90 days were set, respectively. Each group included six samples at each time point. In addition, the fresh blood vessel without any preservation was tested as a control which provided baseline reference data.

#### 2.2.3 Preservation methods

The CSG: preservation solution (100 mL): 50 mL of sterilized NS, 50 mL of medium‐199 culture solution and 100 U of penicillin–streptomycin solution. The acquired abdominal aortas were placed in a 2.5 mL preservation tube containing the above solution and were sealed with a sealing membrane, and then were stored in a refrigerator at 4°C. The preservation solution was changed every 3 days.

The FSG: preservation solution (100 mL): 45 mL of sterilized NS, 45 mL of medium‐199 culture solution, 10 mL of DMSO and 100 U of penicillin–streptomycin solution. The acquired abdominal aortas were placed in a 2.5 mL preservation tube containing the above solution. After being sealed with sealing membranes, the tubes were stored in a freezer at −80°C, and the decreasing rate of temperature was set at 1°C/min. After 2 h of freezing, the samples were immediately transferred to a liquid nitrogen container for further frozen storage.

The ASG: The preservation solution was the same as the CSG. 2 mL of preservation solution and the acquired abdominal aortas were put in a 2.5 mL preservation tube sealed with a sealing membrane and then stored at room temperature (25 ± 2°C).

### 2.3 Detection method

Before the detection, the 2 cm abdominal aorta of each group was cut into five segments of about 0.4 cm in length. Every segment was examined for pathogen culture, vascular structure, cellular activity, vasomotor function and tension resistance detection, respectively.

#### 2.3.1 Aetiology detection

The blood vessel specimens were grinded with an aseptic grinder, and then, the abrasive solution was smeared in the blood plate supplied with Chinese blue Agar medium. The specimens were cultured in an incubator at 35°C for 5 days to observe the growth of bacteria.

#### 2.3.2 Vascular cell activity detection (MTT assay)

To inactivate vascular cells and establish a blank control group, fresh blood vessels should undergo a 10‐min boiling process. Following this, carefully transfer the vascular segment into a 2.5 mL centrifuge tube and add 100 μL of freshly prepared MTT solution (Appendix S1). Incubate the test tube in a shaker at 37°C for 1 h. Subsequently, elute the blue‐purple crystals present within the vascular cells using DMSO. Add 100 μL of the eluent to each well of a 96‐well plate. Utilizing the Multiscan Spectrum, measure the absorbance values (*A*) of the eluent from each preserved group of blood vessels at 570 nm, as well as the absorbance values (*A_0_*) of the blank group solution. Additionally, the mass (*M*) of each group of vascular segments was measured. The MTT value is expressed as the absorbance value per unit weight: *MTT value*=(*A*–*A*_*0*_)/*M*.

#### 2.3.3 Vasoconstriction ability detection

The contractility of blood vessels was evaluated by detecting the response of vascular smooth muscle to norepinephrine. The 7 mL freshly configured HEPES solution was added to each bath of the Powerlab four‐channel physiological recorder. During the whole period, the HEPES solution in the bath was changed every 15 min. When heated to 37°C, the vascular segment of the experimental groups was placed on the tension probe in the bath, and the initial tension was adjusted to 2 mN. After the tension balance, the distance between the two probes was adjusted continuously, with an amplitude of 0.5 mN each time, until the tension balance reached about 4 mN. Then, 7 μL norepinephrine was immediately added, and the tension change (Δ*F*), before and after adding the drug, was recorded.

#### 2.3.4 Histopathological detection

As we previously reported,^13^ vascular samples were fixed with 10% neutral formalin fix solution, and then dehydrated, embedded and sliced. HE staining, Masson staining and EVG staining were performed respectively (Appendix S1). The degeneration of vascular segment intima, collagen fibres and elastic fibres was observed through an optical microscope. To evaluate the preservation effect of different preservation schemes on vascular skeleton structure, pathological staining results were evaluated by two pathologists in a mutually blind manner. We developed a semiquantitative assessment criterion based on endothelial cell detachment, vascular wall oedema and collagen or elastic fibre degeneration. 0 point was recorded when endothelial cells and vascular walls were well preserved, endothelial cell detachment was scored as 1, vessel wall oedema was defined when the gap between the fibrous layers of the inner vessel wall widened more than 1.5 times the normal gap and was scored as 2, and 3 point was recorded when the vessel wall was oedematous with focal degeneration or rupture of collagen fibres or elastic fibres. The higher the score, the more serious the structural damage to the blood vessel wall.

#### 2.3.5 Tension resistance test

6‐0 prolene thread was used to sew a loop at both ends of the vascular segment with the ‘8’ suture method for traction. Then, the loop at one end of the vascular segment was hung on the fixed hook of the tension meter, and another loop was hung on the hook of the tension table. Initially, kept the vessel in a relaxing state, and pressed the ‘zero key’ to remove the influence of gravity on the vascular segment. Then slowly rotated the gear dial of the tension table to make the wire loop gradually tense, and continued to increase the tension until the wire loop was pulled out from the blood vessel segment, and recorded the maximum tension *F*_max_ (*N*) on the tension gauge at this time.

### 2.4 Statistical analysis

Measurement data are expressed by mean ± standard deviation, following a normal distribution, and median (quartile spacing) in non‐normal distribution. For the comparison of measurement data between multiple groups, analysis of variance was used for the normal distribution, while the rank sum test was used for the non‐normal distribution. Comparing the measurement data between the two groups, the *t*‐test was used for the normal distribution, and the rank‐sum test was used for the non‐normal distribution. An error diagram was used to describe the observation index. Differences were considered statistically significant when *p* < 0.05. All data were analysed by spss version 22.0 software (IBM).

## 3 RESULTS

### 3.1 Comparison of general data

**FIGURE 1:**
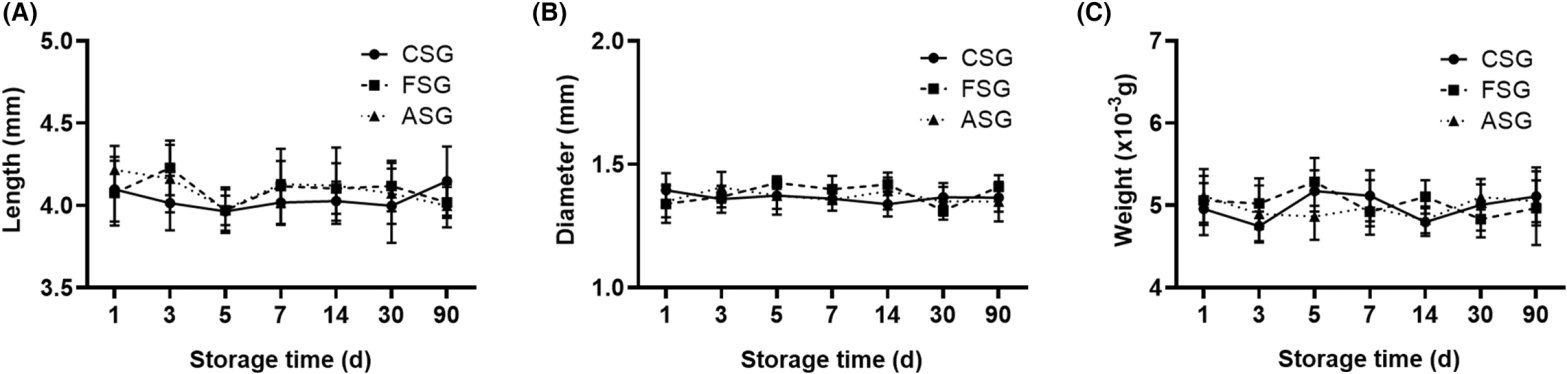
Comparison of general data among different groups. There were no statistically significant differences in the length (A), diameter (B) and weight (C) of blood vessels among the three experimental groups. ASG, ambient storage group (25 ± 2°C); CSG, cold storage group (4°C), also known as low‐temperature refrigeration; FSG, frozen storage group (−196°C), also known as deep hypothermia freezing.

The general data of experimental groups at the same time point, including the length, diameter and weight, were compared in this study (Table 1). The average length, diameter and weight of the fresh blood vessel were 4.16 ± 0.14 mm, 1.34 ± 0.07 mm and (5.10 ± 0.22) × 10^−3^ g. There was no statistical difference in general data of blood vessels among the three experimental groups (Table 1 and Figure 1).

**TABLE 1:** Comparison of general data among different groups.

Item	Group	Storage time (day)						
		1	3	5	7	14	30	90
Length (mm)	CSG	4.10 ± 0.20	4.02 ± 0.17	4.00 ± 0.13	4.02 ± 0.14	4.03 ± 0.12	4.00 ± 0.23	4.15 ± 0.21
	FSG	4.08 ± 0.20	4.23 ± 0.17	3.97 ± 0.09	4.12 ± 0.23	4.10 ± 0.15	4.12 ± 0.15	4.02 ± 0.09
	ASG	4.22 ± 0.15	4.12 ± 0.21	3.98 ± 0.13	4.13 ± 0.14	4.12 ± 0.23	4.07 ± 0.19	4.00 ± 0.13
Diameter (mm)	CSG	1.40 ± 0.07	1.36 ± 0.06	1.37 ± 0.08	1.36 ± 0.01	1.34 ± 0.05	1.37 ± 0.06	1.37 ± 0.06
	FSG	1.34 ± 0.08	1.37 ± 0.04	1.43 ± 0.02	1.40 ± 0.06	1.42 ± 0.03	1.31 ± 0.04	1.41 ± 0.05
	ASG	1.35 ± 0.06	1.41 ± 0.06	1.37 ± 0.05	1.36 ± 0.04	1.40 ± 0.08	1.35 ± 0.06	1.35 ± 0.08
Weight (10^−3^ g)	CSG	5.00 ± 0.32	4.75 ± 0.18	5.18 ± 0.25	5.12 ± 0.31	4.80 ± 0.17	5.01 ± 0.31	5.12 ± 0.35
	FSG	5.06 ± 0.30	5.02 ± 0.31	5.29 ± 0.29	4.92 ± 0.18	5.10 ± 0.20	4.83 ± 0.22	5.00 ± 0.45
	ASG	5.12 ± 0.33	4.90 ± 0.35	4.86 ± 0.28	5.00 ± 0.33	4.82 ± 0.16	5.10 ± 0.15	5.05 ± 0.25

Abbreviations: ASG, ambient storage group (25 ± 2°C); CSG, cold storage group (4°C), also known as low‐temperature refrigeration; FSG, frozen storage group (−196°C), also known as deep hypothermia freezing.

### 3.2 Results of aetiology detection

**FIGURE 2:**
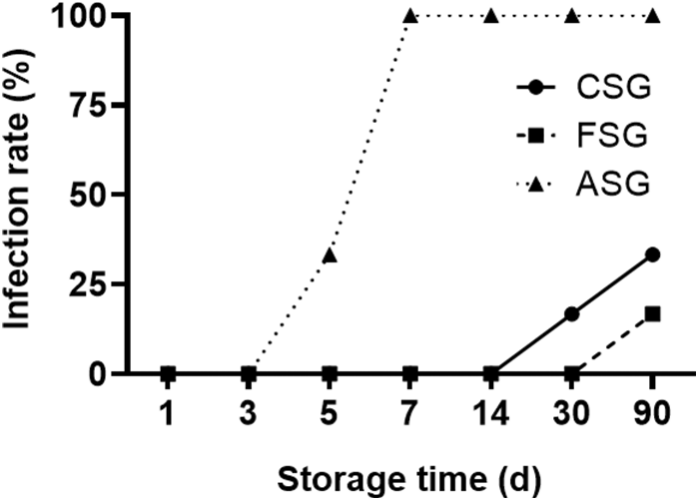
Comparison of vascular infection rate among different groups. ASG blood vessels exhibited a positive pathogen culture starting from the 5th day of preservation. After 7 days of preservation, the pathogen positive rate reached 100%. CSG blood vessels demonstrated a pathogen positive rate of 16.7% after 30 days of preservation, whereas FSG blood vessels did not exhibit any pathogen detection at the 30‐day mark of preservation. ASG, ambient storage group (25 ± 2°C); CSG, cold storage group (4°C), also known as low‐temperature refrigeration; FSG, frozen storage group (−196°C), also known as deep hypothermia freezing.

In ASG, the positive rate of aetiology detection reached 33.3% on the 5th day of preservation, and all samples were gradually contaminated in the following time points of testing. The positive rate of aetiology detection was 16.7% on the 30th day and 33.3% on the 90th day in CSG. In FSG, no pathogenic bacteria were detected in the blood vessels within the 30th day, and the pathogenic positive rate was 16.7% on the 90th day. (Figure 2).

### 3.3 Effect on vascular function of different preservation groups

**FIGURE 3:**
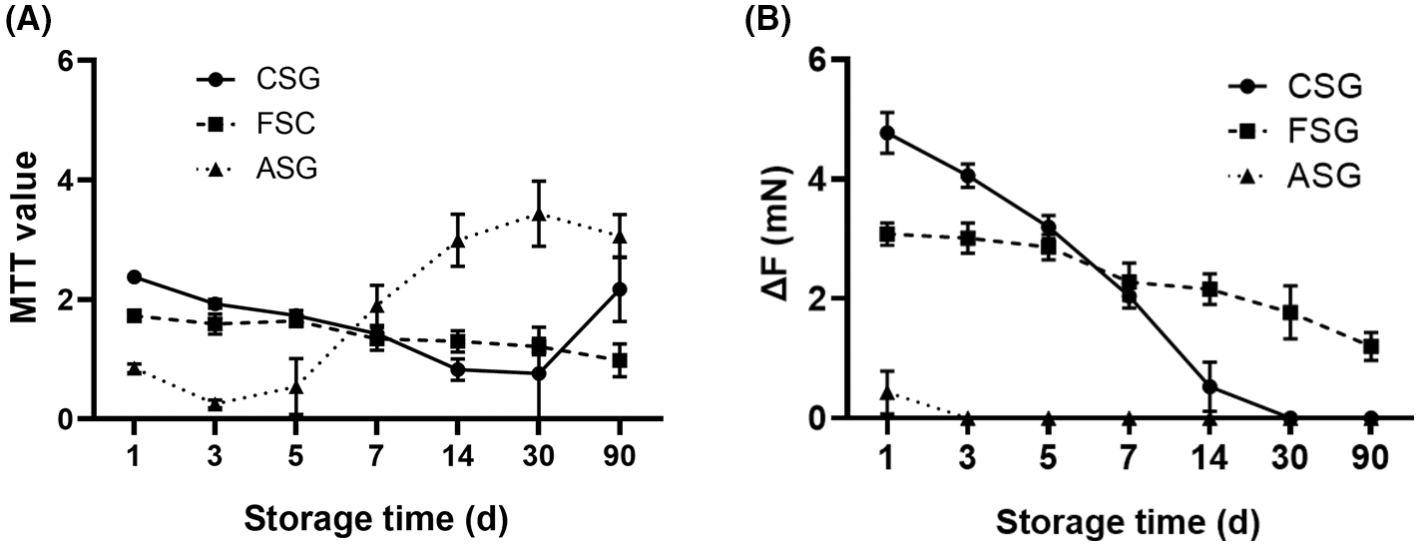
Comparison of vascular function among different groups. (A) Comparison of MTT values among different groups. As the storage time is prolonged, the MTT values of CSG and FSG blood vessels gradually decrease. Surprisingly, ASG blood vessels demonstrate an abnormal increase in MTT values starting from the 5th day of storage, which is considered to be caused by pathogen infection. (B) Comparison of vasoconstriction ability among different groups. As the storage time prolongs, the vasoconstriction ability of each group of blood vessels gradually decreases, with ASG blood vessels showing the most rapid decline, followed by CSG blood vessels in the second place, while FSG blood vessels maintain the longest vasoconstriction ability. ASG, ambient storage group (25 ± 2°C); CSG, cold storage group (4°C), also known as low‐temperature refrigeration; FSG, frozen storage group (−196°C), also known as deep hypothermia freezing.

The activity of vascular cells was evaluated by the MTT test. The MTT value of the fresh vessel was 2.75 ± 0.09. As shown in Table 2 and Figure 3A, the activity of cells of all three experimental groups gradually decreased with the prolonged preservation time. Within the first 3 days of storage, the vascular cell activity in CSG was significantly higher than that in FSG (*p* < 0.001). However, when the storage time exceeds 14 days, the vascular cell activity in FSG was statistically better than that in CSG (*p* = 0.003). Surprisingly, in the ASG, the MTT value decreased gradually in the first 5 days and then increased afterward. Because the pathogenic positive rate in ASG was gradually increased after 5 days of storage (Figure 2), we believe that the abnormal increase of MTT value in ASG was due to bacterial contamination.

**TABLE 2:** Comparison of vascular function among different groups.

Item	Group	Storage time (day)						
		1	3	5	7	14	30	90
MTT (OD/m)	CSG	2.38 ± 0.06	1.93 ± 0.07	1.73 ± 0.08	1.43 ± 0.12	0.83 ± 0.18	0.76 ± 0.77	0.22 ± 0.54
	FSG	1.73 ± 0.06^a^	1.59 ± 0.17^a^	1.64 ± 0.08	1.34 ± 0.19	1.30 ± 0.18^a^	1.21 ± 0.14^a^	0.98 ± 0.28^a^
	ASG	0.85 ± 0.07^a^ ^,^ ^b^	0.25 ± 0.07^a^ ^,^ ^b^	0.54 ± 0.47^a^ ^,^ ^b^	1.9 ± 0.34^a^ ^,^ ^b^	3.00 ± 0.44^a^ ^,^ ^b^	3.44 ± 0.55^a^ ^,^ ^b^	3.06 ± 0.36^a^ ^,^ ^b^
Δ*F* (mN)	CSG	4.78 ± 0.34	4.06 ± 0.20	3.20 ± 0.20	2.04 ± 0.20	0.53 ± 0.41	0	0
	FSG	3.08 ± 0.19^a^	3.01 ± 0.26^a^	2.86 ± 0.21^a^	2.28 ± 0.31^a^	2.16 ± 0.25^a^	1.77 ± 0.44^a^	1.20 ± 0.23^a^
	ASG	0.43 ± 0.36^a^ ^,^ ^b^	0^a^ ^,^ ^b^	0^a^ ^,^ ^b^	0^a^ ^,^ ^b^	0^a^ ^,^ ^b^	0^b^	0^b^

Abbreviations: ASG, ambient storage group (25 ± 2°C); CSG, cold storage group (4°C), also known as low‐temperature refrigeration; FSG, frozen storage group (−196°C), also known as deep hypothermia freezing.

^a^ Comparison with the cold storage group (*p* < 0.05).

^b^ Comparison with the frozen storage group (*p* < 0.05).

The response of vascular smooth muscle to norepinephrine was applied to evaluate vasoconstriction. The vasoconstriction value of the fresh vessel was 6.02 ± 0.08 mN. With the extension of preservation time, the vasoconstriction value of vessels in all experimental groups gradually decreased (Table 2, Figure 3B). The vasoconstriction of ASG was gradually decreasing from the first day and completely disappeared on the 3rd day of storage. In the first 5 days of storage, the vasoconstriction of CSG was higher than that of FSG (*p* = 0.044), but the results were opposite since the 7th day of preservation. Vessels in CSG had completely lost their contraction response to norepinephrine on the 30th day of storage, while vessels in FSG still had a weak response on the 90th day.

### 3.4 Effect of different preservation groups on vascular structure

**FIGURE 4:**
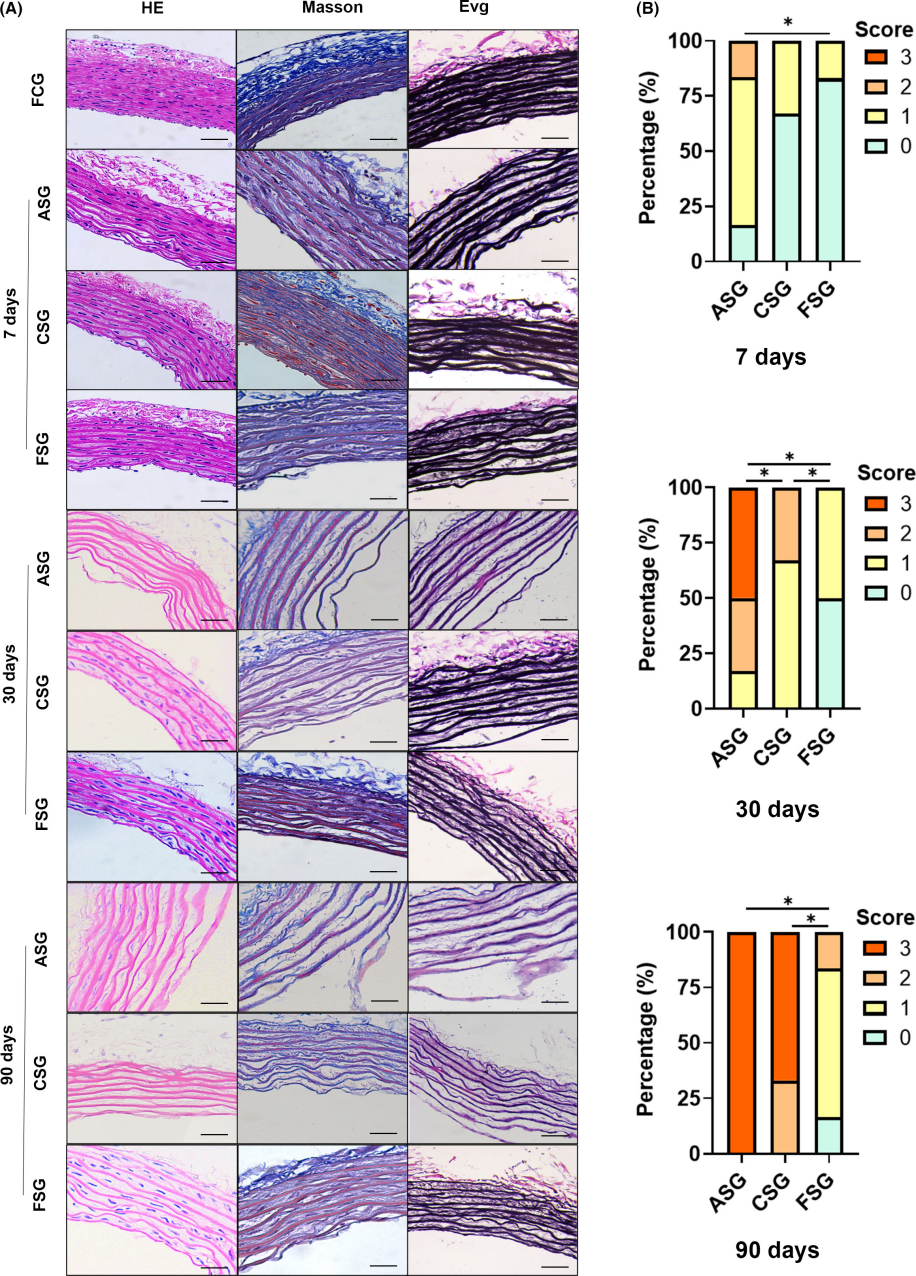
Comparison of vascular structure among different groups. (A) Pathological staining of blood vessels in different groups (×200). In the HE staining of fresh blood vessels, the results revealed the adherence of monolayer endothelial cells to the internal elastic membrane. The vessel wall structure appeared clear, showing no signs of delamination, and exhibiting good continuity in the smooth muscle layer. The nuclei of the cells were distinctly visible. Masson staining demonstrated blue‐coloured collagen fibres within the vessel wall, indicating a well‐preserved and continuous collagen structure. Additionally, in the EVG staining, the elastic fibres appeared as purple‐black, suggesting the presence of intact and continuous elastic fibres within the vessel wall. With the prolonged storage time, As the preservation time increases, blood vessels in each preservation group undergo varying rates and degrees of the following changes: (1) endothelial cell shedding and disappearance, (2) collagen or elastic fibre oedema and (3) collagen or elastic fibre rupture. Scale bar: 50 μm. (B) Histogram of percentage accumulation of pathological scores of different preservation groups. Based on the temporal changes observed in Figure A regarding vascular structure, we have established a semi‐quantitative evaluation standard. A score of 0 is assigned when endothelial cells and blood vessel walls are well‐preserved, while a score of 1 is given when endothelial cells detach. Blood vessel wall oedema is defined as the expansion of the gap between the fibrous layers of the inner wall of the blood vessel to more than 1.5 times the normal gap, and it receives a score of 2. When blood vessel wall oedema is accompanied by focal degeneration or rupture of collagen or elastic fibres, a score of 3 is assigned (*p < 0.05, nonparametric rank‐sum test). ASG, ambient storage group (25 ± 2°C); CSG, cold storage group (4°C), also known as low‐temperature refrigeration; FSG, frozen storage group (−196°C), also known as deep hypothermia freezing.

Pathological staining was performed in each experimental group at the same time point and compared with the fresh vessel which was set as control (Figure 4). The results indicated that, with the prolonged storage time, the changes in vascular wall structure were mainly divided into three stages: (1) endothelial cell shedding and disappearance, (2) collagen or elastic fibre oedema, and (3) collagen or elastic fibre rupture. The change of vascular wall in ASG was most rapid and obvious. On the 7th day of preservation, most of the vascular endothelium in ASG was lost and even collagen and elastic fibre oedema were visible, while the vascular endothelial cells in CSG and FSG remained almost in well condition. On the 30th day of preservation, the vascular endothelial cells in CSG completely disappeared, and some vascular collagen and elastic fibres appeared oedema. While the vascular collagen and elastic fibres in FSG remained in good condition, and vascular endothelial cells were still intact. The vascular changes in ASG were the most obvious, most of the vascular collagen and elastic fibres showed oedema and even fibre breakage. With the storage time extending to 90 days, the vascular collagen and elastic fibres in CSG showed oedema and rupture, and there was no statistical difference compared with the ASG. Although the endothelial cells in FSG almost disappeared, their collagen and elastic fibres remained in good condition. We developed a semiquantitative assessment criterion based on endothelial cell detachment, vascular wall oedema and collagen or elastic fibre degeneration (Figure 4B). There was no statistically significant difference in the pathological scores between CSG and FSG in the first 7 days of storage (*p* = 0.523). When the storage time was more than 30 days, the pathological score of CSG was higher than that of FSG, and the difference was statistically significant (*p* = 0.003).

### 3.5 Comparison of tension resistance

**FIGURE 5:**
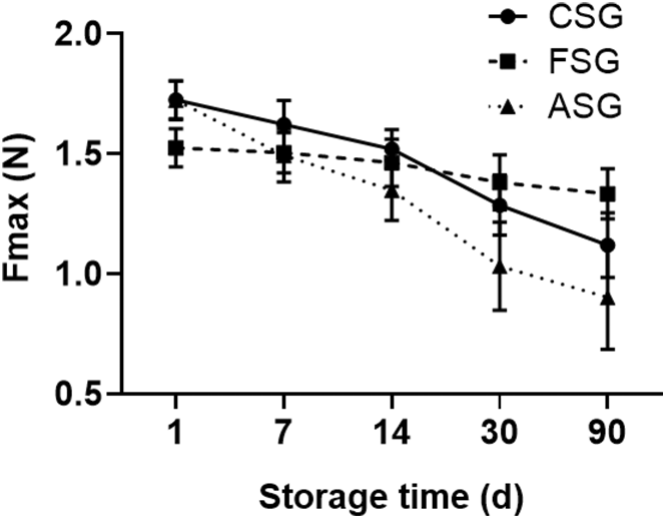
Comparison of vascular tension among different groups. As the duration of storage increases, the ability of each group of blood vessels to tolerate tension diminishes to varying extents. ASG, ambient storage group (25 ± 2°C); CSG, cold storage group (4°C), also known as low‐temperature refrigeration; FSG, frozen storage group (−196°C), also known as deep hypothermia freezing.

The tension value of the fresh blood vessel was 1.72 ± 0.06 N. With the extension of preservation time, the vascular tension gradually decreased in all experimental groups. Vessels in CSG and ASG had higher tension values than that FSG in the first 5 days of preservation (*p* = 0.009). Between the 7th and 30th day, there was no statistical difference in the three experimental groups. When the storage time is extended to 90 days, the tension value of vessels in FSG was significantly higher than that in CSG (*p* = 0.023) and ASG (*p* = 0.006) (Table 3, Figure 5).

**TABLE 3:** Comparison of ultimate tension of blood vessels among different groups.

Group	Storage time (day)						
	1	3	5	7	14	30	90
CSG	1.72 ± 0.08	1.71 ± 0.07	1.64 ± 0.09	1.62 ± 0.10	1.52 ± 0.08	1.29 ± 0.13	1.12 ± 0.14
FSG	1.52 ± 0.08^a^	1.52 ± 0.07^a^	1.50 ± 0.07^a^	1.50 ± 0.08	1.46 ± 0.10	1.38 ± 0.11	1.33 ± 0.11^a^
ASG	1.72 ± 0.08^b^	1.66 ± 0.10^b^	1.56 ± 0.08	1.49 ± 0.11	1.35 ± 0.13^a^	1.03 ± 0.18^a^ ^,^ ^b^	0.90 ± 0.22^a^ ^,^ ^b^

Abbreviations: ASG, ambient storage group (25 ± 2°C); CSG, cold storage group (4°C), also known as low‐temperature refrigeration; FSG, frozen storage group (−196°C), also known as deep hypothermia freezing.

^a^ Comparison with the cold storage group (*p* < 0.05).

^b^ Comparison with the frozen storage group (*p* < 0.05).

## 4 DISCUSSION

With the rapid development of vascular surgical techniques and organ transplantation, vascular reconstruction is gradually gaining popularity in clinical practice, prompting an increasing demand for vascular grafts. Allogeneic vessels are excellent vascular graft that requires a scientific preservation scheme to protect their structures and functions. Under the current situation of donor shortage, the optimal preservation condition of allogeneic vessels is more and more important. Although there are many storage methods for blood vessels, it is of great value, for clinicians, to discover the effect of different preservation schemes on the structure and function of allogeneic vessels.

On account that the cell metabolism could be suppressed under low temperatures, cold storage is applied to preserve the cells and tissues. A study has confirmed that cell metabolism could be almost completely arrested under the deep cryogenic condition with −196°C, which could keep the cell activity for the long term.^14^ O'Brien, et al.^15^ first reported the deep cryogenic storage of cardiovascular tissues in 1975. After comparing the clinical outcome of aortic valve replacement with fresh aortic valves and cryopreserved aortic valves, they argued that the activity of allogeneic valves could be kept for a long time under deep cryogenic conditions. In the following years, the technique of deep cryogenic storage continued greatly improving and the preservative effect was gradually satisfying. It has been widely applied to the long‐term storage of cells and tissue in recent years.^16^, ^17^ However, deep hypothermia freezing is not the most perfect preservation scheme for allografts. The intracellular freezing and solution effects are the two main reasons for cellular damage during cooling and freezing.^18^, ^19^ Contrary to deep cryogenic storage, the aim of cold storage was to keep the metabolism level of cells at a low level which could keep the activity of cells within a certain time, instead of completely stopping the cell metabolism. Cold storage is more promising in the preservation of isolated organs in clinical practice because it is convenient for operation, and does not need complex procedures such as cooling and rewarming, and expensive equipment.^20^, ^21^

Safety is the foremost prerequisite for the clinical application of isolated blood vessels, with the primary concern being the prevention of pathogen growth and reproduction during storage. In this study, we observed that in the ASG, pathogens were detected on the 5th day, and significant damage to the vascular structure was observed by the 7th day, suggesting that the employed protocol failed to effectively preserve allogeneic blood vessels for an extended duration. Conversely, in the CSG and FSG, no pathogens were detected, and the integrity of the vascular wall structure remained relatively intact for up to 30 days. These findings indicate that refrigerated and cryogenic storage techniques can effectively preserve isolated blood vessels.

Appropriate vasodilation and contraction function are crucial for allogeneic blood vessels to withstand the impact of blood flow and maintain hemodynamic stability following vascular reconstruction. The contractile function of blood vessels relies on the activity of smooth muscle cells. Under refrigeration conditions, the absence of complex freezing and rewarming processes eliminates cell damage caused by intracellular freezing and solution effects. Garbe, et al.^22^ have reported that the endothelial‐dependent relaxation function and smooth muscle‐dependent relaxation of isolated blood vessels can be effectively preserved at 4°C. However, at this temperature, cell metabolism cannot be completely inhibited, leading to the gradual accumulation of hypoxic damage in the cells over time. Thus, in addition to preservation temperature and solution, the preservation time becomes another crucial factor that influences the effectiveness of blood vessel preservation. Our research findings indicate that as the storage time increases, the activity of vascular smooth muscle cells gradually diminishes, leading to a decline in the contractile function of the blood vessels. Interestingly, during the initial 5 days of preservation, the CSF group's blood vessels exhibited stronger cell activity and contraction function compared to those of the FSG group. We attribute this disparity to cell damage caused by the cooling and rewarming processes involved in the preservation of the FSG group's blood vessels. Therefore, for ultra‐short‐term storage lasting <5 days, simple static refrigeration proves more advantageous than deep low‐temperature freezing.

A complete vascular structure and optimal mechanical performance are essential prerequisites for a vascular substitute and crucial factors that directly impact the success or failure of surgery. Our research findings indicate that as the storage time increases, the changes in the vascular wall structure can be categorized into three stages: endothelial cell detachment and disappearance, wall oedema, and disintegration and breakage of collagen or elastic fibres. Among these changes, endothelial cells are particularly susceptible to damage during preservation. Starting from the 7th day of storage, all groups exhibited varying degrees of necrosis and shedding of vascular endothelial cells. Therefore, future research efforts should focus on enhancing preservation methods and solutions to protect vascular endothelial cells, as they hold significant importance in maintaining the integrity and function of the vascular substitute. In comparison with the ASG group, both the CSG and FSG groups demonstrated a predominantly intact vascular wall structure even after 30 days of storage. This indicates that both simple static refrigeration and deep low‐temperature freezing can effectively preserve the vascular wall structure within a 30‐day timeframe. Furthermore, the results of mechanical performance tests revealed that when the storage time was <30 days, the mechanical performance of the CSG group's blood vessels was comparable to, and in some cases even superior to, that of the FSG group. However, when the storage time exceeded 90 days, the mechanical performance of the CSG group was significantly lower than that of the FSG group. Consequently, when the preservation duration for isolated blood vessels extends beyond 30 days, deep low‐temperature freezing is the recommended preservation method.

Undoubtedly, the influence of aging on the vascular system is widely acknowledged.^23^ As individuals advance in age, notable transformations take place in the vasculature. These alterations encompass structural modifications, such as the thickening and stiffening of blood vessel walls, along with functional changes characterized by diminished elasticity and impaired vasodilation capacity.^24^, ^25^ Regrettably, our study did not delve into the specific differences in arteries between young and elderly mice. However, investigating age‐dependent changes in arteries would undoubtedly offer a more comprehensive understanding of vascular substitutes and provide valuable insights into strategies aimed at manipulating them. Besides, it should be noted that allogeneic vessels might be recognized as foreign objects when applied in surgeries. On account that several antigens could be formed by endothelial cells, therefore the rejection to allogeneic vessels mainly occurs in the endothelium of vessels. Kadner, et al.^26^ and Feingold, et al.^27^ both reported a lower expression level of ABO blood group antigens in isolated vessels preserved in frozen storage conditions compared with the freshly isolated vessel. In our study, we observed that the storage of isolated blood vessels for more than 7 days resulted in varying degrees of necrosis and shedding of endothelial cells. However, the implications of this process on vascular immunogenicity and postoperative patency necessitate further in vivo experimental research for a comprehensive understanding. In summary, our study offers valuable insights into the selection of in vitro vascular preservation protocols and provides a foundation for future research endeavours in this field.

## 5 CONCLUSION

In conclusion, both cold storage and frozen storage could effectively preserve the structure, tension resistance, vasomotor function and activity of vascular cells in isolated vessels. Cold storage has an advantage over frozen storage when preserved within 7 days, while frozen storage is significantly better than cold storage after 14 days.

## AUTHOR CONTRIBUTIONS

**Zhang‐Yong Ren:** Conceptualization (equal); data curation (equal); formal analysis (equal); investigation (equal); methodology (equal); resources (equal); software (equal); visualization (equal); writing – original draft (equal); writing – review and editing (equal). **Qiao Wu:** Conceptualization (equal); data curation (equal); formal analysis (equal); investigation (equal); methodology (equal); resources (equal); software (equal); visualization (equal); writing – original draft (equal); writing – review and editing (equal). **Bing Pan:** Data curation (equal); formal analysis (equal); investigation (equal); methodology (equal); resources (equal); software (equal). **Jia‐Zong Liu:** Data curation (equal); formal analysis (equal); investigation (equal); methodology (equal); software (equal); visualization (equal). **Qiang He:** Data curation (equal); formal analysis (equal); resources (equal); software (equal); supervision (equal); writing – original draft (equal); writing – review and editing (equal). **Ren Lang:** Conceptualization (equal); funding acquisition (equal); methodology (equal); project administration (equal); supervision (equal); validation (equal); writing – original draft (equal); writing – review and editing (equal). **Shao‐Cheng Lyu:** Conceptualization (equal); data curation (equal); formal analysis (equal); investigation (equal); methodology (equal); project administration (equal); resources (equal); software (equal); supervision (equal); validation (equal); visualization (equal); writing – original draft (equal); writing – review and editing (equal).

## CONFLICT OF INTEREST STATEMENT

No benefits in any form have been received or will be received from a commercial party related directly or indirectly to the subject of this article.

## CONSENT FOR PUBLICATION

Final approval of manuscript: All authors.

## Supporting information


Appendix S1:

## Data Availability

The data supporting this study's findings are available from the corresponding authors.
