# Supplementary material for: Effects of different preservation schemes on isolated rat artery
Source: J Cell Mol Med. 2023 Jun 25;27(16):2362–71. doi: 10.1111/jcmm.17822 (PMC10424285; doi:10.1111/jcmm.17822)
Supplement: Supplementary file 1 — Appendix S1: [file JCMM-27-2362-s001.docx]

**Supplementary information**

**DMSO:** Dimethyl sulfoxide. DMSO is a polar aprotic solvent used in chemical reactions, polymerase chain reactions, and as a cryoprotectant vitrification agent for the preservation of cells, tissues, and organs. DMSO is used in cell freezing media to protect cells from ice crystal-induced mechanical injury. It is used for frozen storage of primary, sub-cultured, and recombinant heteroploid and hybridoma cell lines, embryonic stem cells, and hematopoietic stem cells.

**MTT:** The MTT assay is used to measure cellular metabolic activity as an indicator of cell viability, proliferation, and cytotoxicity. This colorimetric assay is based on the reduction of a yellow tetrazolium salt (3-(4,5-dimethylthiazol-2-yl)-2,5-diphenyltetrazolium bromide or MTT) to purple formazan crystals by metabolically active cells. The viable cells contain NAD(P)H-dependent oxidoreductase enzymes which reduce the MTT to formazan. The insoluble formazan crystals are dissolved using a solubilization solution and the resulting-colored solution is quantified by measuring absorbance at 500-600 nanometers using a multi-well spectrophotometer. The darker the solution, the greater the number of viable, metabolically active cells.

**EVG staining:** The Verhoeff stain, also known as the Verhoeff-van Gieson stain, is a histological staining procedure developed by Frederick Herman Verhoeff in 1908. EVG staining is one of the most commonly-used stains to visualize elastic tissue, as found in blood vessel walls, elastic cartilage, lungs, skin, bladder, and some ligaments.

**HE staining:** Hematoxylin and eosin stain is one of the principal tissue stains used in histology. It is the most widely used stain in medical diagnosis and is often the gold standard. H&E is the combination of two histological stains: hematoxylin and eosin. The hematoxylin stains cell nuclei a purplish blue, and eosin stains the extracellular matrix and cytoplasm pink, with other structures taking on different shades, hues, and combinations of these colors. Hence, we can easily differentiate between the nuclear and cytoplasmic parts of a cell, and additionally, the overall patterns of coloration from the stain show the general layout and distribution of cells and provides a general overview of a tissue sample's structure.

**Masson staining:** Masson's trichrome is a stain that highlights collagen fibers. This method is very popular and is used in histology to differentiate collagen and muscle fibers on tissue sections.
